# Effects of structured and unstructured interventions on fundamental motor skills in preschool children: a meta-analysis

**DOI:** 10.3389/fpubh.2024.1345566

**Published:** 2024-06-27

**Authors:** Delong Chen, Guanggao Zhao, Jinmei Fu, Sunli Shun, Liqiang Su, Zihao He, Ruiming Chen, Tianle Jiang, Xuewen Hu, Yunong Li, Fanchao Shen

**Affiliations:** ^1^School of Physical Education, Nanchang University, Nanchang, China; ^2^Jiangxi Sports Science and Medicine Center, Nanchang, China; ^3^Physical Education College, Jiangxi Normal University, Nanchang, China; ^4^School of Sports and Human Sciences, Beijing Sport University, Beijing, China

**Keywords:** structured, unstructured, fundamental motor skills, preschool children, meta-analysis

## Abstract

**Background:**

It has been suggested that higher levels of fundamental motor skills (FMS) promote the physical health of preschool-aged children. The impacts of structured and unstructured interventions on FMS in children aged 10–16 years have been widely acknowledged in previous studies. However, there is a lack of relevant studies in preschool-aged children.

**Objective:**

This meta-analysis aimed to compare the effects of structured and unstructured interventions on FMS in preschool-aged children.

**Methods:**

The PubMed, Web of Science, and Google Scholar databases were searched from inception to 1 November 2023 to identify experiments describing structured and unstructured interventions for FMS in preschool-aged children. The Downs and Black Checklist was used to assess the risk of bias. A random effects model was used for the meta-analysis to evaluate the pooled effects of interventions on FMS. Subgroup analyses based on the duration and characteristics of the intervention were conducted to identify sources of heterogeneity.

**Results:**

A total of 23 studies with 4,068 participants were included. There were 12 studies examining structured interventions, 9 studies examining unstructured interventions, and 6 studies comparing structured vs. unstructured interventions. The risk of bias in the included studies was generally low. All interventions significantly improved FMS in preschool-aged children compared to control treatments (*p* < 0.05). Structured interventions had more significant effects on locomotor skills (LMSs) in preschool-aged children than unstructured interventions (Hedges’ *g* = 0.44, *p* = 0.04). The effects of structured interventions were strongly influenced by the total intervention duration, such that long-term interventions were more effective (Hedge’s *g* = 1.29, *p* < 0.001).

**Conclusion:**

Structured interventions play a crucial role in enhancing FMS among young children, especially when considering LMSs. These interventions require consistent and repeated practice over time to reach proficiency.

**Systematic review registration:**

PROSPERO, identifier number CRD42023475088, https://www.crd.york.ac.uk/prospero/display_record.php?ID=CRD42023475088.

## 1 Introduction

Fundamental motor skills (FMS) are the basis for children to perform more complex movements (1). Furthermore, achieving FMS can ensure normal development and help to maintain health (2). FMS are usually divided into locomotor skills (LMS) (e.g., running and jumping) and object control skills (OCS) (e.g., catching and throwing) (1). In addition, FMS are closely related to the maintenance of physical activity levels (3), physical fitness (4), overweight and obesity (5), rapid brain development and neuromuscular maturity (6), cognitive and social development (2), and other developmental indicators. FMS are considered the foundation for an active lifestyle (7).

FMS among children—especially jumping ability—have declined over a 13-year period, according to a trend survey (8). Moreover, 9.2% of children exhibited below-average FMS scores (9), with lower FMS scores observed in poverty areas compared to low-poverty areas (10, 11). Because early school age is considered a window of opportunity for developing FMS (12), it is important to promote FMS during the first years of life (13). While all children develop a rudimentary fundamental motor pattern over time, mature patterns of FMS do not develop naturally (14). Implementing effective FMS interventions in school-aged children helps maintain a healthy level and reduces the risk of future adverse conditions.

Currently, the World Health Organization (WHO) (15), the National Association for Sport and Physical Education (NASPE) (16), and organizations such as the National Physical Activity Plan Alliance (NPAPA) (17) emphasize the importance of structured and unstructured activities for preschool-aged children to achieve health requirements. Structured interventions include school physical education (PE), school/club sports programs, and active after-school care. Unstructured interventions include active travel, active play, and informal games. Previous studies have shown that structured interventions are more effective than unstructured interventions in terms of improving FMS in girls aged 10–16 years (18).

Previous meta-analyses focused solely on summarizing the effects of either structured or unstructured interventions on FMS in preschool-aged children. Notably, studies have examined the impact of PE on FMS in preschool-aged children (19) and the influence of active play on FMS (20). However, no meta-analyses have examined both structured and unstructured interventions. While some experimental studies have compared these structured and unstructured interventions in preschool settings, they have yielded inconsistent results (21, 22). Therefore, it is necessary to consolidate and analyze the effects of existing structured and unstructured interventions on FMS in preschool-aged children.

This meta-analysis examined the impacts of structured and unstructured interventions on the development of FMS in preschool-aged children. This research aimed to establish a foundational understanding for future targeted health promotion interventions.

## 2 Methods

The study was performed in accordance with the Preferred Reporting Items for Systematic Review and Meta-Analysis (PRISMA) guidelines (23). The inclusion/exclusion criteria and analytical methods were specified and registered in PROSPERO (http://www.crd.york.ac.UK/PROSPERO) before the study was initiated (PROSPERO reference number CRD42023475088).

### 2.1 Search strategy

The PubMed, Web of Science, and Google Scholar electronic databases were searched from inception to 1 November 2023. The search strategy was developed based on the eligibility criteria and outcomes of interest. In addition, the bibliographies of all eligible original papers and reviews were manually searched. The search terms were as follows: ‘fundamental motor skills’, ‘gross motor skills’, ‘locomotor skills’, ‘object* skills’, and ‘young children’. The search strategies and results returned for each database are shown in the Supplementary Table S1. Two researchers independently identified relevant articles by screening titles and reviewing abstracts (CDL and CRM). Two reviewers (CDL and CRM) examined the full texts of the articles for eligibility.

### 2.2 Inclusion and exclusion criteria

We developed the following inclusion criteria in accordance with the PICOS approach (24): (1) participants: preschoolers (2–6 years of age); (2) interventions: any type of structured or unstructured intervention aimed at increasing FMS; (3) control group: usual child care or kindergarten classes or another intervention strategy; (4) outcome: inclusion of FMS test metrics (total FMS score [total FMS], LMS, and OCS); (5) study design: an intervention trial with an intervention duration longer than 4 weeks (randomized controlled trial [RCT], cluster randomized controlled trials [CRT], or comparative studies in which the sample is randomized, and non-RCTs).

The exclusion criteria were as follows: (1) literature not published in Chinese or English; (2) reviews or studies missing key data; (3) non-controlled studies; and (4) non-controlled trials.

### 2.3 Data extraction

One author (CDL) extracted the following information from each eligible study: study background (name of the first author, year, and study location), sample characteristics (number of participants, age of participants, and number of girls and boys), design [intervention (RCT or non-RCT)], and instruments used to assess FMS outcomes. We also recorded the number of weeks of intervention, the duration and frequency of the interventions, and the descriptions of the interventions.

### 2.4 Criteria for risk of bias assessment

Two researchers (CRM and JTL) individually evaluated the risk of bias for each eligible study. Any disagreements were resolved through a consensus meeting. To determine the interrater agreement for the risk of bias assessment, the percentage agreement between the evaluators (CRM and JTL) was calculated. SPSS software version 26.0 (IBM Corporation, Armonk, NY, United States) was used to calculate the intraclass correlation coefficient (ICC) analysis.

Two independent researchers (CRM and JTL) assessed the full studies for bias using the Downs and Black checklist (25). The Downs and Black Checklist was used to evaluate the risk of bias for non-randomized and randomized control trials. The checklist consists of 27 items. The majority of questions were rated as either “yes” (= 1) or “unable to determine/no” (= 0), except for item five, which was rated on a 3-point scale (yes = 2, partial = 1, and no = 0). The maximum score was 32, with higher scores indicating better quality. The quality of the studies was classified as excellent (≥26), good (18–23), fair (13–17), or poor (≤14).

### 2.5 Meta-analysis of intervention studies

A meta-analysis was performed using STATA 15.0 statistical software (produced by Stata Corp, https://www.stata.com/). When different studies used different measurement methods and tools, Hedges’ g and 95% CI were used to measure the effect size. According to Cohen’s (1988) classification of effect sizes, a value less than 0.2 indicates a small effect, a value between 0.2 and 0.79 indicates a medium effect, and a value greater than or equal to 0.80 indicates a large effect (26). Calculations were performed using the Cochrane Handbook method for assessing the effective sample size for CRT (27). The ICC was estimated to be 0.031 (28). The sample sizes calculated using this method are presented in Supplementary Table S2. The pooled ES of the effect was determined using random effects models. A *p*-value of <0.05 and 95% confidence intervals (CIs) were used as criteria for identifying significant differences. Additionally, to understand the effectiveness of the intervention modality, we compared the changes from baseline to endpoint data between groups. The formulas for the mean and SD from pre- to post-change values were as follows: Mean_change_ = Mean_post_ – Mean_pre_ and SD_change_ = SQRT [(SD_pre_^2^ + SD_post_^2^) – (2 × Corr × SD_pre_ × SD_post_)], with the correlation coefficient set at 0.5 based on the Cochrane Collaboration Handbook guidelines (29). To distinguish the effect errors caused by different clusters, subgroup analyses were performed based on different outcome indicators (total FMS, LMS, and OCS). In multiarm studies (21, 22), the experimental group is compared to the control group in pairs (e.g., in a three-arm study, experimental group 1 is compared to the control group, experimental group 2 is compared to the control group, and experimental group 1 is compared to experimental group 2). Moreover, in the meta-analysis, the sample size of the control group was recorded only once during the statistical analysis to avoid artificially inflating the true sample size (21, 22).

Heterogeneity (i.e., the degree of variation between studies) was determined using the *I*^2^ statistic. *I*^2^ values <25, 50, and 75% were considered to indicate low, moderate, and high heterogeneity, respectively (30). To evaluate the likelihood of publication bias, funnel plots were generated, and Egger’s test was performed.

### 2.6 Subgroup analyses

First, we classified three outcome indicators (total FMS, LMS, and OCS). We subsequently conducted subgroup analyses based on the characteristics of the different interventions. Since structured interventions have a defined dose and duration, we used different intervention durations for the corresponding subgroup analysis. The total intervention duration was divided into three categories: (0, 1,100) min, (1,100, 3,000) min, and (3,000, +∞) min.

Unstructured interventions involve many steps, and according to the characteristics of the intervention, they can be divided into changing the environment, providing official policy guidance, and an unstructured curriculum.

## 3 Results

### 3.1 Selection process

**Figure 1:**
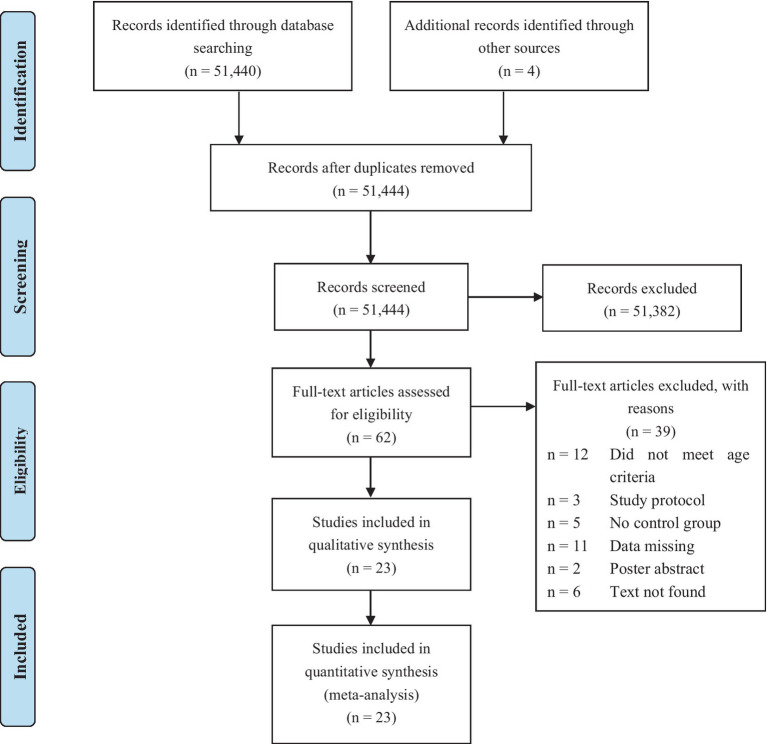
PRISMA flow chart of the search results and articles identified for inclusion.

A total of 51,440 relevant studies were retrieved, and 23 works were ultimately included based on the inclusion and exclusion criteria. The screening process is shown in Figure 1. In total, 14 randomized controlled trials (22, 31–43), eight cluster randomized controlled trials (21, 44–50), and one clinical trial (51) were included in the meta-analysis.

### 3.2 Basic features of the included studies

The 23 studies included in this meta-analysis are described in detail in Table 1. There were 12 structured intervention studies (21, 22, 31, 33, 36, 37, 42, 43, 45, 47, 49, 51), 9 unstructured intervention studies (21, 22, 32, 35, 40, 41, 44, 46, 48), and 5 structured vs. unstructured intervention studies (21, 22, 34, 38, 39, 50), 2 of which were 3-arm trials (21, 22). The sample characteristics were included as follows: 23 studies involved a total of 4,068 preschool-aged children aged 3–6 years, with sample sizes ranging from 27 to 648. Two studies focused on preschoolers at risk e.g., exposure to biological risks (chronic diseases, genetic diseases, etc.), or environmental risks (single parenthood, poverty, etc.) (34, 35); one study focused on malnourished preschoolers (41); and one study involved only a sample of girls (38); the rest of studies included normal, healthy samples. The results characteristics were included as follows: 17 studies provided total FMS data (21, 22, 31–33, 37, 41–51), 9 studies provided LMS data (21, 32, 39, 40, 46–49, 51), and 12 studies provided OCS data (21, 32, 34–36, 38, 40, 46–49, 51). A total of 9 of the 17 studies reporting total FMS included both LMS scores and OCS scores. Exposures and comparison conditions were described as follows: the majority of studied interventions had a duration ranging from 8 to 24 weeks, and two studies included interventions longer than 24 weeks (25 weeks (51) and 36 weeks (44)). Notably, the structured intervention studies included in this meta-analysis described the intervention doses and the intervention durations ranged from 300 to 7,200 min. Among the unstructured intervention studies, five included details regarding intervention doses, with durations ranging from 300 to 21,600 min (21, 22, 32, 35, 41). In four studies (40, 44, 46, 48), due to the intervention involving the physical environment, space, and other factors, the dose could not be estimated. Intervention intensity was not reported in all studies; for example, participants were required to maintain intensity markers such as 50–80% of the maximum heart rate during the intervention.

**Table 1:** Basic characteristics of the included studies.

Degree of bias	Study, year, country	Case/*n* (age range; %girls); Design	Weeks of intervention; description; duration; frequency; content of intervention	FMS indicators; Instrument
Good	Minghetti (51), Switzerland	I: 26/C: 22 (4–6 years; 50.0% girls); non-RCT	25; structure vs. control; 45 min; 1 d/w; I: structured FMS intervention: Perform FMS training, including balance, control, and movement exercises C: uphold daily habits	Total FMS; LMS; OCS TGMD-2
Good	Bonvin (44), Switzerland	I: 313/C: 335 (3–5 years; 48.3% girls); CRT	36; unstructured vs. control; data not shown; I: workshops for educators and parents; flyers; documentation and support at childcare centers through coordinators; financial support for childcare centers to design activity-friendly spaces C: regular curriculum	Total FMS ZNA
Fair	Venetsanou (31), Greece	I: 28/C: 38 (59.79 ± 6.4 months; 45.5% girls); RCT	20; structure vs. control; 45 min; 2 d/w; I: structured dance course: a combination of music/movement elements, singing games and dances, and the development of coordination abilities (kinesthetic differentiation, balance ability, orientation in space, rhythmic ability, and response ability) C: regular curriculum	Total FMS MOT 4–6
Good	Reilly (45), UK	I: 268/C: 277 (3–6 years; 49.9% girls); CRT	24; structure vs. control; 30 min; 3 d/w; I: nursery element—PA lessons; home element—health education leaflets C: regular curriculum	Total FMS MABC
Fair	Palmer (32), United States	I: 30/C: 16 (3–5 years; 59% girls); RCT	15; unstructured vs. control; 30 min; 3 d/w; I: Motor skills At Playtime (MAP): setting 3–4 motor skill stations on the playground, children could select if they wanted to engage in the motor skill stations and use the motor skill equipment for non-station-specific play C: regular curriculum	Total FMS; LMS; OCS TGMD-3
Fair	Branje (46), Canada	I: 104/C: 93 (3–5 years; 44.7% girls); CRT	24; unstructured vs. control; data not shown; I: outdoor play with loose parts: loose parts kit includes bucket and lid, rope and pulley, etc. They would always be available to the children C: regular curriculum	Total FMS; LMS; OCS TGMD-3
Fair	Adamo (47), Canada	I: 40/C: 43 (3–5 years; 53.0% girls); CRT	24; structure vs. control; 60–90 min; 5 d/w; I: booster sessions: PA and FMS intervention courses were conducted by professional instructors C: regular curriculum	Total FMS; LMS; OCS TGMD-2
Good	Bellows (33), United States	I: 132/C: 131 (3–5 years; 45% girls); RCT	18; structure vs. control; 15–20 min; 4 d/w; I: structured PA: includes multiple activities focusing on one or a group of skills from one of the three gross motor skill categories (balance, LMS, and OCS) C: regular curriculum	Total FMS PDMS-2
Fair	Robinson (34), United States	I: 38/C: 40 (3–5 years; 43.6% girls); RCT	9; structure vs. unstructured; 30 min; 2 d/w; I: structured FMS intervention: motor skill instruction for OCS (24 min), closure activity (2–3 min) C: unstructured free time	OCS TGMD-2
Good	Roach (21), Canada	I: 16/C: 19 (3–5 years; 42.9% girls); CRT	8; structure vs. control; 45 min; 2 d/w; I: Skill-based: four FMS sites were rotated, and demonstrations were guided by professionals C: free play	Total FMS; LMS; OCS TGMD-2
Good	Roach (21), Canada	I: 16/C: 19 (3–5 years; 54.3% girls); CRT	8; unstructured vs. control; 45 min; 2 d/w; I: planned active play: using a bank of age-appropriate games developed to improve FMS C: free play	Total FMS; LMS; OCS TGMD-2
Good	Roach (21), Canada	I: 16/I: 16 (3–5 years; 50.0% girls); CRT	8; structure vs. unstructured; 45 min; 2 d/w; I1: Skill-based I2: planned active play	Total FMS; LMS; OCS TGMD-2
Good	Hardy (48), Australia	I: 263/C: 167 (3–5 years; 49.8% girls); CRT	20; unstructured vs. control; data not shown; I: workshop for preschool staff; resources for preschools; contact with health promotion professionals C: provides books on sun and road safety	Total FMS; LMS; OCS TGMD-2
Fair	Hamilton (35), United States	I: 12/C: 15 (3–5 years; 40.7% girls); RCT	8; unstructured vs. control; 45 min; 2 d/w; I: parent-assisted lessons: train parents and involve them in the lessons C: regular curriculum	OCS TGMD
Fair	Wasenius (49), Canada	I: 59/C: 62 (3–5 years; 40.5% girls); CRT	24; structure vs. control; 60 min; 5 d/w; I: workshops for childcare providers; a structured activity program including FMS training and preschool activity programming C: regular curriculum	Total FMS; LMS; OCS TGMD-2
Fair	Tortella (22), Italy	I: 62/C: 36 (4–6 years; 49.0% girls); RCT	10; structure vs. control; 30 min; 1 d/w; I: structured activity: activities are carried out according to the sequence of specific activity areas, and the instructor provides the use of activity equipment C: regular curriculum	Total FMS MABC-2
Fair	Tortella (22), Italy	I: 43/C: 36 (4–6 years; 49.4% girls); RCT	10; unstructured vs. control; 30 min; 1 d/w; I: free play C: regular curriculum	Total FMS MABC-2
Fair	Tortella (22), Italy	I: 62/I: 43 (4–6 years; 55.1% girls); RCT	10; structure vs. unstructured; 30 min; 1 d/w; I1: structured activity I2: free play	Total FMS MABC-2
Good	Morgan (36), Australia	I: 61/C: 64 (3–5 years; 39.2% girls); RCT	10; structure vs. control; 70 min; 1 d/w; I: 55 min of father-led structured FMS training in the workshop and 15 min of training at home C: uphold daily habits	OCS TGMD-3
Good	Jones (43), Australia	I: 77/C: 73 (3–5 years; 43.3% girls); RCT	24; structure vs. control; 20 min; 3 d/w; I: structured lesson: focused on one gross motor skill in each lesson and have unstructured time after class to practice current learning skills C: regular curriculum	Total FMS TGMD-2
Fair	Jones (50), Australia	I: 52/C: 45 (3–5 years; N/A girls); CRT	20; structure vs. unstructured; 20 min; 3 d/w; I: structured PA: lessons focusing on one motor competency each week C: outside for free play	Total FMS TGMD-2
Fair	Mostafavi (37), Iran	I: 30/C: 45 (4–6 years; N/A girls); RCT	8; structure vs. control; 30 min; 3 d/w; I: structured gymnastic lessons C: regular curriculum	Total FMS TGMD-2
Good	Veldman (38), United States	I: 38/C: 16 (3–5 years; 100% girls); RCT	9; structure vs. unstructured; 30 min; 2 d/w; I: structured FMS lessons: focusing on OCS and targeted six ball skills C: outdoor free play	OCS TGMD-2
Good	Alhassan (39), United States	I: 43/C: 28 (3–5 years; 50.7% girls); RCT	24; structure vs. unstructured; 30 min; 5 d/w; I: structured PA: lessons focusing on one of the skills of the TGMD-2 LMS C: unstructured free play time	LMS TGMD-2
Good	Trost (40), Australia	I: 17/C: 17 (3–6 years; 50.0% girls); RCT	8; unstructured vs. control; data not shown; I: novel digital application: digital active games. The game is designed to be fun and focus on specific motor skills C: uphold daily habits	LMS; OCS TGMD-2
Good	Abessa (41), United States	I: 170/C: 169 (36–69 months; 46.0% girls); RCT	12; unstructured vs. control; Stage 1: 240 min/d, 1 month; Stage 2: 20–40 min; 1 d/w, 2 months; I: access to the playroom and provided with stimulation and play materials, Stage 1: play-based motor, language, and personal-social activities; Stage 2: 8–10 games aimed at gross motor activities and different types of sensory-motor training. C: routine medical care	Total FMS Denver II-Jimma
Fair	Yin (42), United States	I: 118/C: 69 (3–5 years; N/A girls); RCT	18; structure vs. control; 30–45 min; 5 d/w; I: outdoor lessons: including gross motor skills, teaching, and dance instruction C: regular curriculum	Total FMS LAP-3

I, intervention; C, control; RCT, randomized controlled trial; CRT, cluster randomized controlled trial; TRT, translational randomized trial; min, minutes; d, day; w, week; total FMS, total fundamental motor skill score; LMSs, locomotor skills; OCS, object control skills; TGMD-2, the test of gross motor development–2nd edition; ZNA, Zurich neuromotor assessment; MOT 4–6, the motoriktest für vier-bis sechsjährige Kinder; TGMD-3, The test of gross motor development–3rd edition; MABC, movement and activity battery in children; PDMS-2, peabody developmental motor scales–2nd edition; MABC-2, movement and activity battery in children–2nd edition; LAP-3, learning achievement profile version 3.

### 3.3 Meta-analysis results

The results of this study are described in detail in Table 2. As shown in Supplementary Figure S1 and in the forest plots for all comparisons, the included study interventions had significant effects on the total FMS, LMS, and OCS in preschool-aged children (*p* < 0.05). The structured intervention was more effective than the unstructured intervention in improving the LMS (*p* < 0.05). No significant publication bias was detected based on Egger’s test for total FMS (*p* = 0.10), LMS (*p* = 0.10), or OCS (*p* = 0.06). The funnel plot is shown in Supplementary Figures S2–S4.

**Table 2:** Subgroup analyses on the effect of intervention on FMS in preschool children.

Outcome	Potential modifiers	Studies, *n*	Effect size (95% CI)	*I*^2^ (%)	*P*-value heterogeneity
Total FMS	**Experimental** vs.**Control**	18	0.35 (0.20 to 0.50)	68.9	<0.001
	**Structured** vs.**Control**	11	0.50 (0.27 to 0.72)	72.3	<0.001
	Total duration				
	<1,100 min	4	0.56 (0.36 to 0.75)	0.3	<0.001
	1,100–3,000 min	5	0.24 (0.10 to 0.38)	0	0.001
	>3,000 min	2	1.29 (0.92 to 1.66)	0	<0.001
	**Unstructured** vs.**Control**	7	0.13 (0.02 to 0.23)	0	0.02
	Trait				
	Environment	2	0.03 (−0.24 to 0.31)	0	0.81
	Official policy	2	0.09 (−0.06 to 0.24)	6.9	0.24
	Curriculum	3	0.24 (0.04 to 0.43)	0	0.02
	**Structured** vs.**Unstructured**	3	0.28 (−0.04 to 0.60)	0	0.08
LMS	**Experimental** vs.**Control**	9	0.69 (0.30 to 1.07)	80.4	<0.001
	**Structured** vs.**Control**	4	0.99 (0.19 to 1.79)	85.1	0.02
	Total duration				
	<1,100 min	1	0.96 (0.14 to 1.78)	–	–
	1,100–3,000 min	1	−0.07 (−0.65 to 0.50)	–	–
	>3,000 min	2	1.53 (1.14 to 1.92)	0	<0.001
	**Unstructured** vs.**Control**	5	0.34 (0.13 to 0.55)	18.2	0.002
	Trait				
	Environment	1	0.25 (−0.10 to 0.60)	–	–
	Official policy	1	0.19 (−0.04 to 0.43)	–	–
	Curriculum	3	0.68 (0.28 to 1.08)	0	0.001
	**Structured** vs.**Unstructured**	2	0.44 (0.03 to 0.86)	0	0.04
OCS	**Experimental** vs.**Control**	11	0.54 (0.22 to 0.87)	77.7	0.001
	**Structured** vs.**Control**	5	0.57 (0.16 to 0.97)	66.2	0.01
	Total duration				
	<1,100 min	2	0.80 (0.46 to 1.13)	0	<0.001
	1,100–3,000 min	1	0.40 (−0.18 to 0.99)	–	–
	>3,000 min	2	0.43 (−0.60 to 1.45)	87.8	0.42
	**Unstructured** vs.**Control**	6	0.55 (0.06 to 1.04)	81.9	0.03
	Trait				
	Environment	1	−0.15 (−0.50 to 0.19)	–	–
	Official policy	1	0.19 (−0.05 to 0.43)	–	–
	Curriculum	4	0.96 (0.11 to 1.82)	79.9	0.03
	**Structured** vs.**Unstructured**	3	2.24 (−0.71 to 5.19)	97.3	0.14

Total FMS, total fundamental motor skills; LMS, locomotor skills; OCS, object control skills.

#### 3.3.1 Total FMS

In total, 16 studies reported total FMS (21, 22, 31–33, 37, 41–49, 51). Compared with the control group, the interventions had a medium effect size (Hedge’s *g* = 0.35, 95% CI = 0.20–0.50, *I*^2^ = 68.9%, *p* < 0.001).

Total FMS data were reported in 11 structured intervention studies (22, 31, 33, 37, 42, 43, 45, 47, 49, 51). Compared with the control group, the structured interventions had a medium-sized effect on FMS (Hedge’s *g* = 0.50, 95% CI = 0.27–0.72, *I*^2^ = 72.3%, *p* < 0.001). Furthermore, when considering the total intervention duration, interventions with a duration of 300–1,100 min (Hedge’s *g* = 0.56) (22, 33, 37), 1,100–3,000 min (Hedge’s *g* = 0.24) (31, 42, 43, 45, 51), and 3,000–7,200 min (Hedge’s *g* = 1.29, 49, 51) had significant effects on FMS in preschool-aged children. There was a low level of heterogeneity (*I*^2^ < 25%).

Total FMS data were reported from six unstructured intervention studies (22, 32, 41, 44, 46, 48). Compared with the control group, the unstructured interventions had a small-sized effect on FMS (Hedge’s *g* = 0.13, 95% CI = 0.02–0.23, *I*^2^ = 0%, *p* = 0.02). Subgroup analysis based on intervention characteristics indicated that only the unstructured curriculum (Hedge’s *g* = 0.24) (21, 32, 41) had a significant effect on the preschool total FMS (*p* = 0.02). There was no heterogeneity between studies (*I*^2^ = 0%).

Three studies comparing structured vs. unstructured interventions reported total FMS data (21, 22, 50). There was no significant difference in the total FMS score between the structured intervention and unstructured intervention groups (*p* = 0.08).

#### 3.3.2 LMS

Eight studies reported LMS data (21, 32, 40, 46–49, 51). Compared with the control group, the interventions had a medium-sized effect on LMS (Hedge’s *g* = 0.69, 95% CI = 0.30–1.07, *I*^2^ = 80.4%, *p* < 0.001).

Four structured intervention studies reported LMS data (21, 47, 49, 51). Compared with the control group, the structured interventions had a medium-sized effect on LMS (Hedge’s *g* = 0.99, 95% CI = 0.19–1.79, *I*^2^ = 85.1%, *p* = 0.02). Subgroup analysis based on the total duration of the intervention revealed that interventions with a duration greater than 3,000 min (Hedge’s *g* = 1.53) (47, 49) had a significant effect on LMS in preschool-aged children. There was no heterogeneity between studies (*I*^2^ = 0%).

Five unstructured intervention studies reported LMS data (21, 32, 40, 46, 48). Compared to the control group, the unstructured interventions had a medium-sized effect on LMS (Hedge’s *g* = 0.34, 95% CI = 0.13–0.55, *I*^2^ = 18.2%, *p* = 0.002). Subgroup analysis based on intervention characteristics indicated that only the unstructured curriculum (Hedge’s *g* = 0.68) (21, 32, 40) had a significant effect on LMS in preschool-aged children (*p* < 0.001). There was no heterogeneity between studies (*I*^2^ = 0%).

Two studies comparing structured and unstructured interventions reported LMS data (21, 39). Compared with unstructured interventions, structured interventions had a medium-sized effect on LMS in preschool-aged children (Hedge’s *g* = 0.44, 95% CI = 0.03–0.86, *I*^2^ = 0%, *p* = 0.04).

#### 3.3.3 OCS

*n* total, 11 studies reported OCS data (21, 32, 35, 36, 40, 46–49, 51). Compared with the control group, the interventions had a medium-sized effect on OCS (Hedge’s g = 0.54, 95% CI = 0.22–0.87, *I*^2^ = 77.7%, *p* = 0.001).

Five structured intervention studies reported OCS data (21, 36, 47, 49, 51). Compared with the control group, the structured interventions had a medium-sized effect on OCS (Hedge’s *g* = 0.57, 95% CI = 0.16–0.97, *I*^2^ = 66.2%, *p* = 0.01). Subgroup analysis based on the total intervention duration revealed that interventions with a total duration of less than 1,100 min (Hedge’s *g* = 0.80) (21, 36) had a significant effect on OCS in preschool-aged children. There was no heterogeneity between studies (*I*^2^ = 0%).

Six unstructured intervention studies reported OCS data (21, 32, 35, 40, 46, 48). Compared with the control group, unstructured interventions had a medium-sized effect on OCS (Hedge’s g = 0.55, 95% CI = 0.06–1.04, *I*^2^ = 81.9%, *p* = 0.03). Subgroup analysis based on intervention characteristics revealed that only the unstructured curriculum (Hedge’s g = 0.96) (21, 32, 35, 40) had a significant effect on OCS in preschool-aged children (*p* = 0.03). There was a high degree of heterogeneity between studies (*I*^2^ = 79.9%).

Three structured vs. unstructured studies reported OCS data (21, 34, 38). There was no significant difference between the structured and the unstructured intervention in terms of OCS in preschool-aged children (*p* = 0.14).

### 3.4 Risk of bias assessment

The two researchers exhibited a high degree of consistency regarding the risk of bias assessment (ICC = 0.78). Overall, seven RCTs showed a fair risk of bias (22, 31, 32, 34, 35, 37, 42). All four CRTs showed a fair risk of bias (46, 47, 49, 50). Furthermore, the non-RCTs showed a good risk of bias (51). Details of the risk of bias assessment are shown in Supplementary Table S3.

## 4 Discussion

The included studies explored the effects of various interventions [childcare environment (52), teacher leadership (19), parental involvement (53), etc.] on FMS indicators in preschool-aged children. However, the use of various interventions leads to a high risk of heterogeneity. Therefore, using a more macro perspective (structured vs. unstructured interventions) may be a feasible approach. This study attempted to analyze trials of structured and unstructured interventions targeting FMS indicators in preschool-aged children. The effects of the structured and unstructured interventions on the FMS index of preschool-aged children were compared. After analyzing 19 intervention trials, we found that after active intervention, the FMS of preschool-aged children can be significantly improved (total FMS: Hedge’s *g* = 0.35; LMS: Hedge’s *g* = 0.69; OCS: Hedge’s *g* = 0.54). This study is consistent with previous studies showing that interventions targeting FMS in preschool-aged children are beneficial (54). Relevant reports show that the level of FMS mastery is still low in preschool-aged children (8, 10, 11). Both structured and unstructured interventions improved FMS in preschool-aged children. However, preschool is the period during which children develop FMS (1, 12). Selecting more efficient intervention measures for this age group is still a challenge among scholars. Similarly, there are no relevant reports on whether structured intervention or unstructured intervention is the better choice for improving the FMS in preschool-aged children. This study is the first known meta-analysis to investigate the effects of structured vs. unstructured interventions on the development of FMS in preschool-aged children.

### 4.1 Effects of structured and unstructured interventions on total FMS in preschool-aged children

This study examined 11 structured intervention studies to determine their effect on FMS in preschool-aged children. The results showed that structured interventions had a significant positive impact on the total FMS in preschool-aged children (Hedge’s g = 0.50, 95% CI = 0.27–0.72, *I*^2^ = 72.3%, *p* < 0.001). The significance of adult leadership within structured interventions lies in its capacity to deliver verbal cues, thereby influencing children’s motor behavior (55). In structured classes, it is essential to provide not only positive feedback but also negative feedback, verbal instructions, demonstrations, and effective organization of practice. The synthesis of this series of functions ultimately fosters the refinement of children’s motor skills (56, 57). Previous studies have suggested that the number of intervention sessions per week is the main reason for the heterogeneity of structured intervention effects (3 per week) (19). However, in this study, the duration of interventions was used as the segmentation point. The results showed that intervention duration and effect size had a “√” type relationship. That is, interventions with a duration of less than 3,000 min (Hedge’s *g* = 1.29) had the largest effect size, followed by interventions lasting more than 1,100 min (Hedge’s *g* = 0.56), and those lasting between 1,100 and 3,000 min (Hedge’s *g* = 0.24) had the smallest effect size. All the studies conducted had low heterogeneity. This may be attributed to the learning characteristics of preschool-aged children. Young learners exhibit high intrinsic motivation and a strong interest in acquiring new knowledge (58). Nevertheless, repetitive instruction on the same content tends to diminish enthusiasm for learning, leading to reduced compliance. Research suggests that short-duration interventions (<1,100 min) may be more effective than medium-duration interventions (1100–3,000 min). Additionally, the two long-term interventions (7,200 min) in this study were considerably longer than those in the remaining studies (<3,000 min), thus enabling extended practice and skill development among children (47, 49). This phenomenon may explain the greater effect size observed in the long-term intervention group. Consequently, emphasizing the necessity for repetitive teaching, practice, and reinforcement of FMS becomes crucial. These findings are consistent with expert opinions in the field, indicating that FMS need to be actively taught and consistently reinforced since they do not appear to develop naturally and maintain themselves automatically (14, 59).

In addition to structured interventions, seven unstructured intervention studies were included. The results showed that unstructured interventions significantly improved the total FMS (Hedge’s *g* = 0.13, 95% CI = 0.02–0.23, *I*^2^ = 0%, *p* = 0.02) in preschool-aged children. Previous studies did not integrate research on unstructured interventions targeting FMS in preschool-aged children and instead used only single-feature literature or systematic reviews (20, 60). However, consistent with those studies, unstructured interventions have health benefits for preschoolers. Unstructured interventions are considered a potential measure for enhancing children’s physical activity and improving their overall development (61, 62). However, some studies have suggested that unstructured interventions can have significant effects only in small samples (62). However, this study revealed that in unstructured interventions involving a large sample, some studies still showed significant improvement in total FMS in preschool-aged children (41, 44, 48). Thus, sample size is not a major factor in the effect of unstructured interventions. A comparison of the results of the included studies revealed that only exercise interventions directly targeting preschool-aged children could affect total FMS. Teachers and caregivers, providing children with ideological courses, or increasing the content of children’s physical activity environment may have more effects on children’s physical, psychological, and other developmental indicators (63–65). After further analysis, we found that unstructured interventions were distinguished based on intervention characteristics. Only unstructured curriculum interventions had a significant effect on total FMS in preschool-aged children. Research suggests that vigorous active play is a good measure for promoting the development of FMS in preschool-aged children (66). Organizations, such as the WHO (67), explicitly require active play for preschoolers. The reason is that in energetically active play, children can fully run, jump, and play (61). The development of children’s athletic ability is strong. In addition, role-playing between peers can more fully mobilize children’s enthusiasm and enable them to develop in games (68).

By analyzing three studies of structured vs. unstructured interventions, this study revealed that structured interventions were not significantly better than unstructured interventions at improving total FMS in preschool-aged children (*p* = 0.08). Structured interventions are controlled in design, and well-structured, repetitive exercise regimens are often performed. However, it is not yet clear whether the structured interventions will also have the same benefits on other indicators of physical development (69). Moreover, child development indicators are not limited to FMS. Therefore, the use of structured interventions in practice should consider more comprehensive design content. Interventions should avoid focusing too closely on the development of one indicator while neglecting the common development of other health indicators. In contrast, although the effects of unstructured interventions on total FMS in preschool-aged children were not as strong as the effects of structured interventions, the former approach still yielded significant positive effects. Moreover, unstructured interventions have positive effects on many child health indicators (70). The rational use of unstructured interventions is feasible.

### 4.2 Effects of structured and unstructured interventions on LMS and OCS in preschool-aged children

The findings of this study indicate that both structured and unstructured interventions exert significant impacts on the LMS and OCS of preschool-aged children. However, it is imperative to acknowledge the potential influence of the number of included studies and methodological variations between LMS and OCS research on the obtained results. Prior research has highlighted that LMSs, such as running and jumping, constitute fundamental components of children’s daily activities (71), and numerous motor interventions, encompassing both structured and unstructured interventions, have been found to promote these skills. This highlights the significant potential for both types of interventions to enhance LMS in preschool-aged children when administered correctly. This study posits that structured interventions outperform unstructured interventions in enhancing LMS in preschool-aged children. This superiority is evident both through direct and indirect comparisons, which consistently indicate that structured interventions yield more favorable outcomes. Furthermore, as proposed by JD Goodway, children’s acquisition of proficient LMS is contingent upon guidance and/or practice (72). In the absence of appropriate guidance, children may fail to master LMS even throughout their adolescent years (73). This highlights the crucial role of structured interventions, particularly guidance, in fostering the development of LMS in preschool children.

On the other hand, the results for control skills, such as throwing and catching, were similar under both structured and unstructured interventions. In indirect comparisons, the effects of structured (Hedge’s *g* = 0.57) and unstructured (Hedge’s *g* = 0.55) interventions compared to the control group were similar. Similarly, direct comparisons did not reveal significant differences (Hedge’s *g* = 2.24, 95% CI = -0.71–5.19, *I*^2^ = 97.3%, *p* = 0.14). This suggests that the development of control skills may require a more complex intervention design (34). The current study found that the allocation of time in structured curricula may be one of the factors influencing the development of OCS. Niko S. Wasenius’s study allocated more intervention time to LMS practice, with only 33% of the time devoted to OCS practice (49). Another study provided additional control skills, such as balls and bats (44). Although structured and unstructured interventions produced similar results in promoting overall motor skill performance, only motor skills showed superior results in structured practice compared to unstructured practice.

Other results showed that increasing outdoor amusement equipment did not lead to a significant improvement in FMS in preschool-aged children, which is consistent with the findings of previous research (22, 46, 74). It should be noted that this study included only a few studies on changing the environment, which may not provide a clear explanation. Additionally, the implementation of outdoor amusement equipment varies greatly, and it is crucial to carefully evaluate and design intervention strategies to determine the positive effects of FMS in preschool-aged children (22). Therefore, it cannot be denied that changing the environment has a positive impact on FMS in preschool-aged children, but further research is needed to fully understand the extent of this impact.

### 4.3 Limitations of the study

Although there are several novel findings in this study, there are still several limitations. For example, the studies included in this meta-analysis used different types of motor interventions. In addition, this study only referred to the duration of the intervention and did not refer to the intensity of the intervention. However, there are no clear reports on the intensity of intervention, and the results only provide good guidance on the duration of intervention. Moreover, data, such as physical activity and other related indicators, were not collected in this study, so there was no analysis of how these factors may affect FMS.

## 5 Conclusion

Scientific interventions are necessary to improve FMS in young children. Well-designed structured interventions are more suitable than natural development for the improvement of FMS in young children, especially when considering LMSs. Moreover, FMS in young children need to be practiced repeatedly for a long time to achieve proficiency.

## Data availability statement

The original contributions presented in the study are included in the article/Supplementary material, further inquiries can be directed to the corresponding author/s.

## Author contributions

DC: Writing – original draft. GZ: Writing – review & editing. JF: Writing – review & editing. SS: Writing – review & editing. LS: Writing – review & editing. ZH: Writing – review & editing. RC: Data curation, Writing – review & editing. TJ: Software, Writing – review & editing. XH: Software, Writing – review & editing. YL: Software, Writing – review & editing. FS: Software, Writing – review & editing.
