# Supplementary material for: Effects of structured and unstructured interventions on fundamental motor skills in preschool children: a meta-analysis
Source: Front Public Health. 2024 Jun 27;12:1345566. doi: 10.3389/fpubh.2024.1345566 (PMC11242925; doi:10.3389/fpubh.2024.1345566)
Supplement: Supplementary file 1 [file Table_1.DOCX]

**Electronic Supplementary Material Table**

**Electronic Supplementary Material Table S1: Search terms used for each database with the amount of results returned.**

| **Database** | **Search Terms** | **Results/Papers** |
| --- | --- | --- |
| PubMed | All Fields: (FMS OR motor skill OR locomotor skills OR object* skills OR gross motor quotient) AND (preschool OR young children） | 8080 |
| Web of Science | Title/Keywords/Abstract: (FMS OR motor skill OR locomotor skills OR object* skills OR gross motor quotient) AND (preschool OR young children) | 11960 |
| Google Scholar | All Fields: ‘fundamental motor skills’ OR ‘gross motor skills’ OR ‘locomotor skills’ OR ‘object* skills’ AND ‘physical activity’ AND ‘children’ | 31400 |

**Electronic Supplementary Material Table S2: Effective sample calculation**

| Study | Research type | Samples from the intervention group | Effective of sample size | Mean of the intervention group | SD of the intervention group | Samples from the control group | Effective sample size of the control group | Mean of the control group | SD of the control group | ICC | Set of assignments | Design effect for the trial |
| --- | --- | --- | --- | --- | --- | --- | --- | --- | --- | --- | --- | --- |
| Alice Minghetti 2021 - total FMS | non-RCT | 26 | 26 | 6 | 11.49 | 22 | 22 | 3.9 | 13.63 | NA | NA | NA |
| Alice Minghetti 2021 - LMS | non-RCT | 24 | 24 | 1.8 | 6.6 | 22 | 22 | 2.4 | 9.11 | NA | NA | NA |
| Alice Minghetti 2021 - OCS | non-RCT | 24 | 24 | 4.2 | 7.06 | 22 | 22 | 1.4 | 6.55 | NA | NA | NA |
| Antoine Bonvin 2013 - total FMS | CRT | 280 | 213 | 1.8 | 3.33 | 308 | 234 | 1.7 | 3.27 | 0.031 | 58 | 1.314276 |
| Fotini Venetsanou 2004 - total FMS | RCT | 28 | 28 | 4.26 | 5.95 | 38 | 38 | 1.027 | 5.25 | NA | NA | NA |
| John J. Reilly 2006 - total FMS | CRT | 268 | 182 | 3.6 | 2.46 | 277 | 189 | 3 | 2.61 | 0.031 | 36 | 1.469306 |
| Kara K. Palmer 2020 - total FMS | RCT | 30 | 30 | 0.32 | 3.64 | 16 | 16 | -1.16 | 4.24 | NA | NA | NA |
| Kara K. Palmer 2020 - LMS | RCT | 30 | 30 | 0.42 | 2.19 | 16 | 16 | -1.46 | 1.99 | NA | NA | NA |
| Kara K. Palmer 2020 - OCS | RCT | 30 | 30 | -0.09 | 2.1 | 16 | 16 | 0.14 | 2.73 | NA | NA | NA |
| Karina Branje 2021 - total FMS | CRT | 103 | 77 | 11.06 | 14.22 | 75 | 56 | 11.13 | 13.75 | 0.031 | 16 | 1.344875 |
| Karina Branje 2021 - LMS | CRT | 103 | 77 | 6.29 | 8.37 | 75 | 56 | 4.16 | 8.61 | 0.031 | 16 | 1.344875 |
| Karina Branje 2021 - OCS | CRT | 103 | 77 | 5.56 | 8.04 | 75 | 56 | 6.73 | 7.01 | 0.031 | 16 | 1.344875 |
| Kristi B. Adamo 2016 - total FMS | CRT | 40 | 28 | 4.18 | 4.02 | 43 | 30 | -1.53 | 3.95 | 0.031 | 6 | 1.428833 |
| Kristi B. Adamo 2016 - LMS | CRT | 40 | 28 | 1.41 | 0.77 | 43 | 30 | 0.21 | 0.75 | 0.031 | 6 | 1.428833 |
| Kristi B. Adamo 2016 - OCS | CRT | 40 | 28 | -0.12 | 0.58 | 43 | 30 | -0.68 | 0.57 | 0.031 | 6 | 1.428833 |
| Laura L. Bellows 2013 - total FMS | RCT | 122 | 122 | 5.73 | 9.12 | 122 | 122 | -0.5 | 8.45 | NA | NA | NA |
| Lindsay Roach 2018a - total FMS | CRT | 16 | 12 | 2.45 | 0.43 | 19 | 14 | 0.31 | 8.61 | 0.031 | 3 | 1.361667 |
| Lindsay Roach 2018a - LMS | CRT | 16 | 12 | 2.5 | 2.01 | 19 | 14 | 0.64 | 1.75 | 0.031 | 3 | 1.361667 |
| Lindsay Roach 2018a - OCS | CRT | 16 | 12 | 1.06 | 1.8 | 19 | 14 | -0.52 | 1.61 | 0.031 | 3 | 1.361667 |
| Lindsay Roach 2018b - total FMS | CRT | 16 | 12 | 2.33 | 0.57 | 19 | 14 | 0.31 | 8.61 | 0.031 | 3 | 1.361667 |
| Lindsay Roach 2018b - LMS | CRT | 16 | 12 | 1.69 | 1.78 | 19 | 14 | 0.64 | 1.75 | 0.031 | 3 | 1.361667 |
| Lindsay Roach 2018b - OCS | CRT | 16 | 12 | 2.37 | 1.54 | 19 | 14 | -0.52 | 1.61 | 0.031 | 3 | 1.361667 |
| Louise L. Hardy 2010 - total FMS | CRT | 207 | 177 | 4.7 | 10.21 | 127 | 109 | 2.8 | 9.53 | 0.031 | 61 | 1.169738 |
| Louise L. Hardy 2010 - LMS | CRT | 207 | 177 | 2.1 | 6.75 | 127 | 109 | 0.8 | 6.55 | 0.031 | 61 | 1.169738 |
| Louise L. Hardy 2010 - OCS | CRT | 207 | 177 | 2.8 | 5.9 | 127 | 109 | 1.7 | 5.7 | 0.031 | 61 | 1.169738 |
| Michelle Hamilton 1999 - OCS | RCT | 12 | 12 | 3.37 | 2.72 | 15 | 15 | -0.06 | 1.81 | NA | NA | NA |
| Niko S. Wasenius 2017 - total FMS | CRT | 44 | 38 | 13.1 | 2.45 | 48 | 41 | 10.3 | 2.14 | 0.031 | 18 | 1.158444 |
| Niko S. Wasenius 2017 - LMS | CRT | 35 | 31 | 9.2 | 1.97 | 48 | 42 | 6.8 | 1.21 | 0.031 | 18 | 1.142944 |
| Niko S. Wasenius 2017 - OCS | CRT | 35 | 31 | 3.4 | 1.13 | 48 | 42 | 3.5 | 1.15 | 0.031 | 18 | 1.142944 |
| Patrizia Tortella 2022a - total FMS | RCT | 42 | 42 | 6.5 | 12.2 | 36 | 36 | 1.73 | 12.67 | NA | NA | NA |
| Patrizia Tortella 2022b - total FMS | RCT | 43 | 43 | 3 | 12.93 | 36 | 36 | 1.73 | 12.67 | NA | NA | NA |
| Philip J. Morgan 2022 - OCS | RCT | 61 | 61 | 4.7 | 5.66 | 64 | 64 | 0.3 | 5.6 | NA | NA | NA |
| Rachel A. Jones 2016 - total FMS | RCT | 77 | 77 | 2.8 | 5.17 | 73 | 73 | 1.1 | 5.43 | NA | NA | NA |
| Reza Mostafavi 2013 - total FMS | RCT | 30 | 30 | 3.1 | 0.9 | 45 | 45 | 2.9 | 0.11 | NA | NA | NA |
| Stewart G. Trost 2020 - LMS | RCT | 17 | 17 | 3 | 6.47 | 17 | 17 | -0.6 | 6.42 | NA | NA | NA |
| Stewart G. Trost 2020 - OCS | RCT | 17 | 17 | 3.3 | 6.52 | 17 | 17 | -2.6 | 6.47 | NA | NA | NA |
| Teklu G. Abessa 2019 - total FMS | RCT | 170 | 170 | 0.98 | 3.33 | 169 | 169 | 0.36 | 2.29 | NA | NA | NA |
| Zenong Yin 2011 - total FMS | RCT | 66 | 66 | 9.06 | 8.24 | 69 | 69 | 8.75 | 10.88 | NA | NA | NA |
| **structure vs. unstructured** |  |  |  |  |  |  |  |  |  |  |  |  |
| Leah E. Robinson 2009 - OCS | RCT | 38 | 38 | 23.33 | 7.38 | 40 | 40 | 0.38 | 4.55 | NA | NA | NA |
| Lindsay Roach 2018c - LMS | CRT | 16 | 12 | 2.5 | 2.01 | 16 | 12 | 1.69 | 1.78 | 0.031 | 3 | 1.330667 |
| Lindsay Roach 2018c - OCS | CRT | 16 | 12 | 1.06 | 1.8 | 16 | 12 | 2.37 | 1.54 | 0.031 | 3 | 1.330667 |
| Lindsay Roach 2018c - total FMS | CRT | 16 | 12 | 2.45 | 0.43 | 16 | 12 | 2.33 | 0.57 | 0.031 | 3 | 1.330667 |
| Patrizia Tortella 2022c - total FMS | RCT | 42 | 42 | 6.5 | 12.2 | 43 | 43 | 3 | 12.93 | NA | NA | NA |
| Rachel A. Jones 2011 - total FMS | CRT | 77 | 23 | 2.8 | 5.17 | 73 | 22 | 1.1 | 5.43 | 0.031 | 2 | 3.325 |
| Sanne L. Veldman 2016 - OCS | RCT | 38 | 38 | 23.87 | 6.65 | 16 | 16 | 0.94 | 4.15 | NA | NA | NA |
| Sofiya Alhassan 2012 - LMS | RCT | 43 | 43 | 22.8 | 19.39 | 28 | 28 | 13.6 | 21.13 | NA | NA | NA |

Note: RCT, randomized controlled trial; CRT, cluster randomized controlled trial; NA, Not applicable

**Electronic Supplementary Material Table S3: Risk of bias assessment**

| Degree of bias | Study, year, country | Research type | Reporting (11) | External Validity (3) | Internal Validity (7) | Selection (6) | Power (5) | Score (32) |
| --- | --- | --- | --- | --- | --- | --- | --- | --- |
| Good | Alice Minghetti 2021, Swiss | non-RCT | 10 | 3 | 5 | 4 | 0 | 22 |
| Fair | Fotini Venetsanou 2004, Greek | RCT | 5 | 3 | 5 | 2 | 0 | 15 |
| Fair | Kara K. Palmer 2020, USA | RCT | 5 | 3 | 5 | 2 | 0 | 15 |
| Good | Laura L. Bellows 2013, USA | RCT | 8 | 3 | 6 | 5 | 0 | 22 |
| Fair | Leah E. Robinson 2009, USA | RCT | 6 | 3 | 6 | 4 | 0 | 19 |
| Fair | Michelle Hamilton 1999, USA | RCT | 5 | 3 | 6 | 4 | 0 | 18 |
| Fair | Patrizia Tortella 2022a, Italy | RCT | 7 | 3 | 5 | 3 | 0 | 18 |
| Fair | Patrizia Tortella 2022b, Italy | RCT | 7 | 3 | 5 | 3 | 0 | 18 |
| Fair | Patrizia Tortella 2022c, Italy | RCT | 7 | 3 | 5 | 3 | 0 | 18 |
| Good | Philip J. Morgan 2022, Australia | RCT | 8 | 3 | 6 | 6 | 0 | 23 |
| Good | Rachel A. Jones 2016, Australia | RCT | 7 | 3 | 6 | 5 | 0 | 21 |
| Fair | Reza Mostafavi 2013, Iran | RCT | 6 | 3 | 5 | 5 | 0 | 19 |
| Good | Sanne L. Veldman 2017, USA | RCT | 6 | 3 | 6 | 5 | 0 | 20 |
| Good | Sofiya Alhassan 2012, USA | RCT | 6 | 3 | 6 | 6 | 0 | 21 |
| Good | Stewart G. Trost 2021, Australia | RCT | 7 | 3 | 6 | 5 | 0 | 21 |
| Good | Teklu G. Abessa 2019, USA | RCT | 9 | 3 | 6 | 6 | 0 | 24 |
| Fair | Zenong Yin 2012, USA | RCT | 7 | 3 | 5 | 3 | 0 | 18 |
| Good | Antoine Bonvin 2013, Swiss | CRT | 7 | 3 | 6 | 6 | 0 | 22 |
| Good | John J. Reilly 2006, UK | CRT | 7 | 3 | 6 | 5 | 0 | 21 |
| Fair | Karina Branje 2022, Canada | CRT | 6 | 3 | 5 | 5 | 0 | 19 |
| Fair | Kristi B. Adamo 2016, Canada | CRT | 5 | 3 | 5 | 3 | 0 | 16 |
| Good | Lindsay Roach 2018a, Canada | CRT | 7 | 3 | 5 | 5 | 0 | 20 |
| Good | Lindsay Roach 2018b, Canada | CRT | 7 | 3 | 5 | 5 | 0 | 20 |
| Good | Lindsay Roach 2018c, Canada | CRT | 7 | 3 | 5 | 5 | 0 | 20 |
| Good | Louise L. Hardy 2010, Australia | CRT | 9 | 3 | 6 | 6 | 0 | 24 |
| Fair | Niko S. Wasenius 2018, Canda | CRT | 7 | 3 | 5 | 4 | 0 | 19 |
| Fair | Rachel A. Jones 2011, Australia | CRT | 7 | 3 | 5 | 4 | 0 | 19 |

Note: RCT, randomized controlled trial; CRT, cluster randomized controlled trial

**
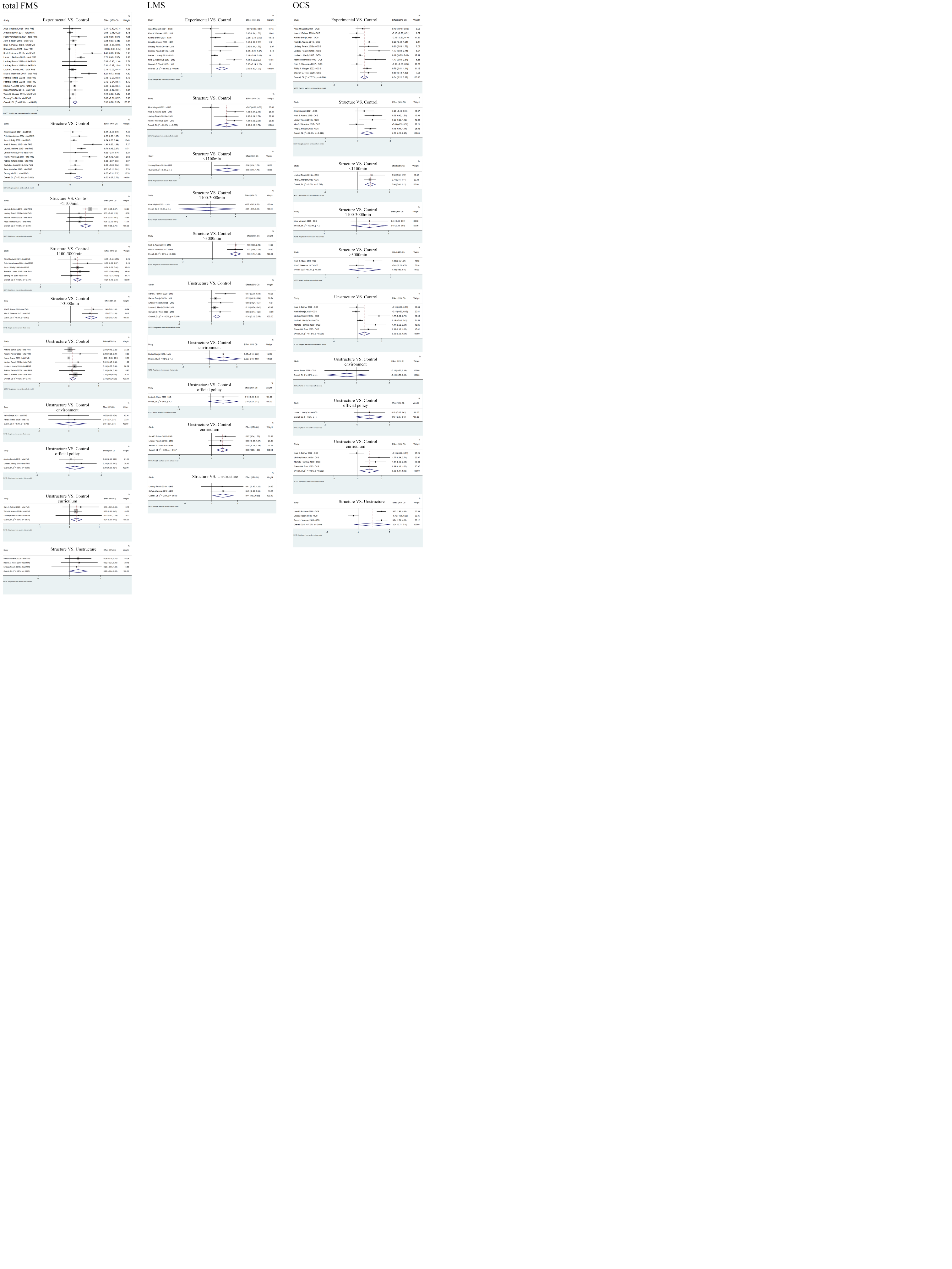
Electronic Supplementary Material Figures**

**Electronic Supplementary Material Figure S1: Forest plots and summary results of the meta-analyses for total FMS, LMS, and OCS**

**
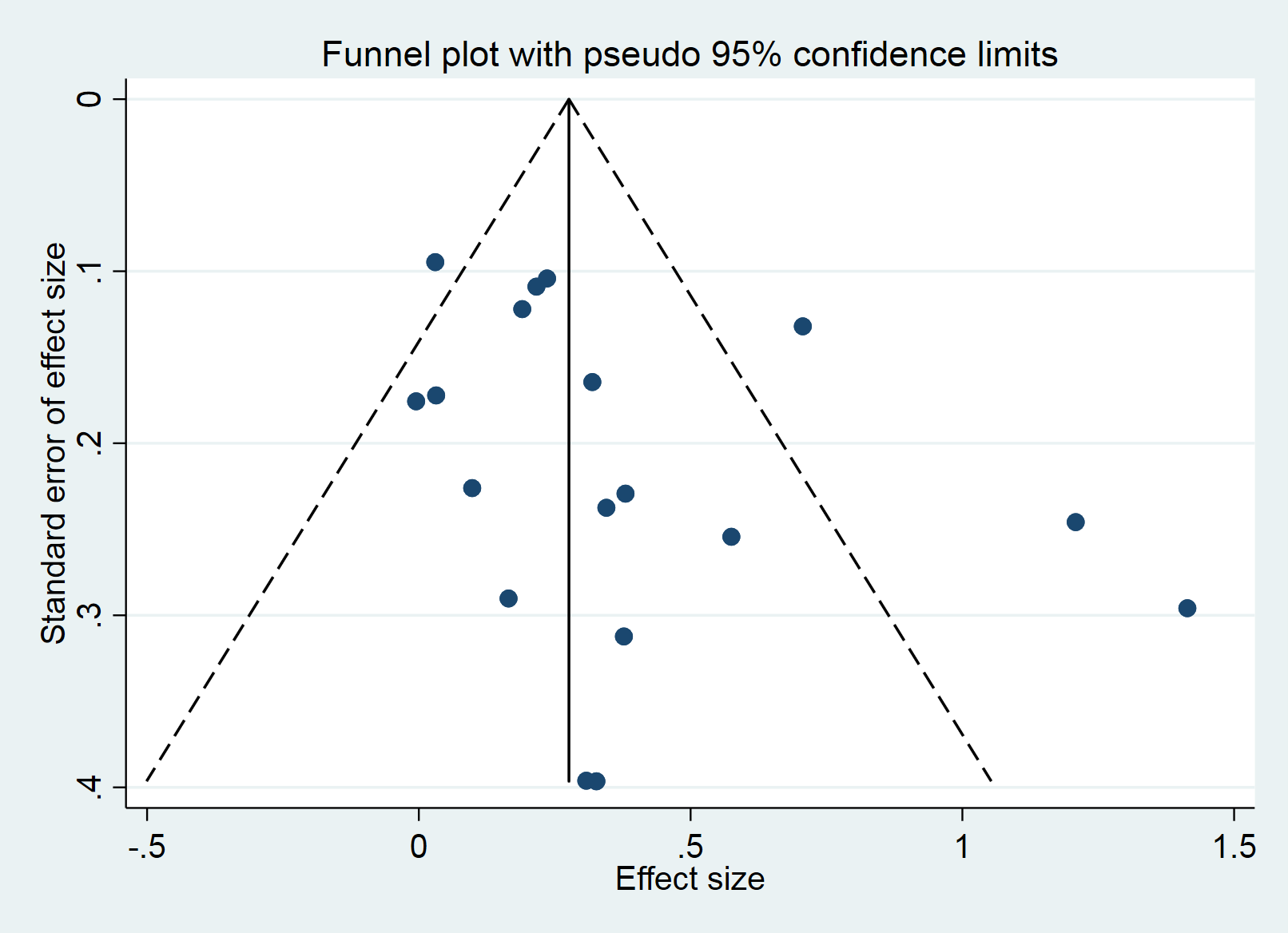
**

**Electronic Supplementary Material Figure S2: Funnel plot of intervention versus control on total FMS**

**
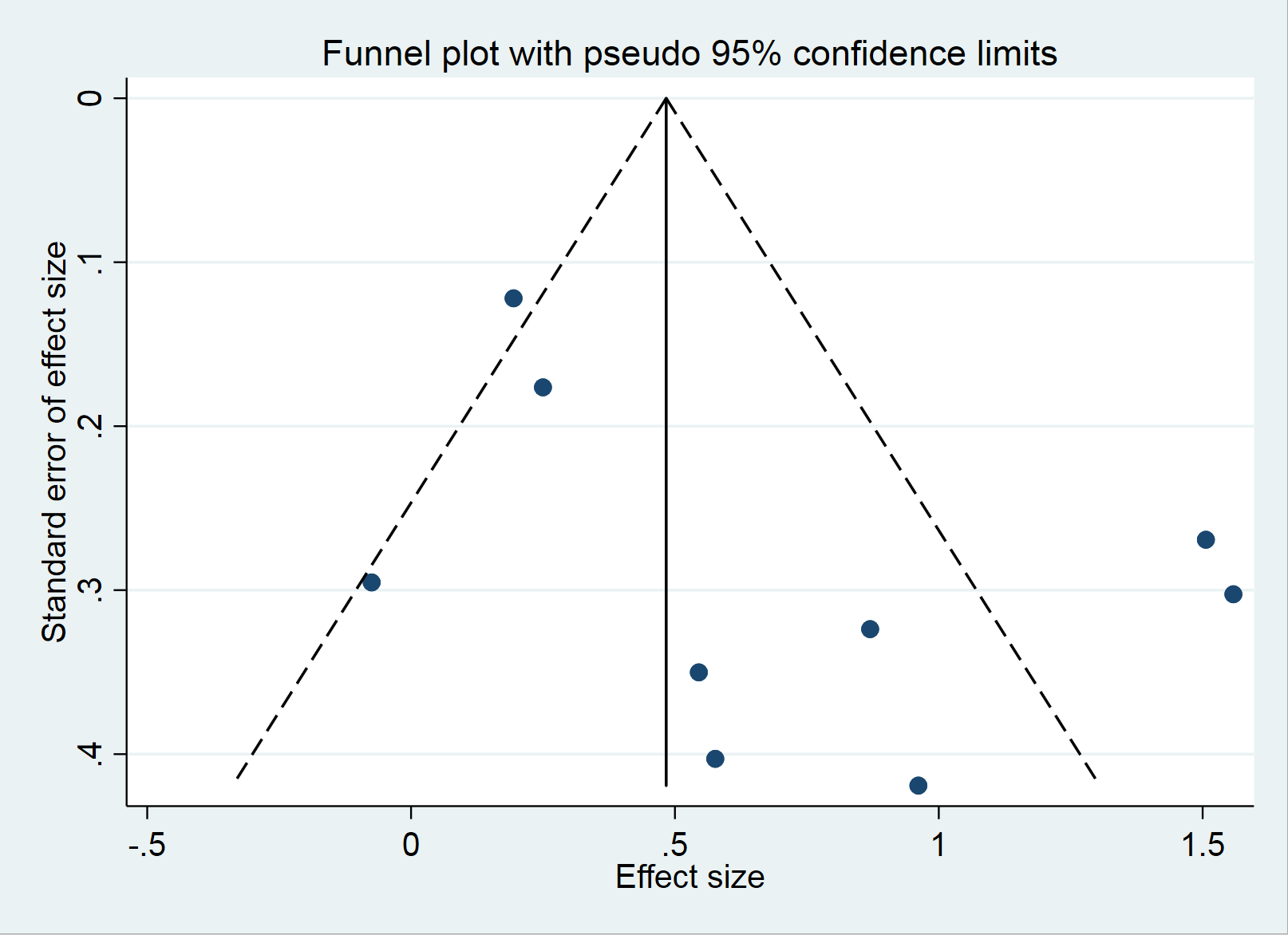
**

**Electronic Supplementary Material Figure S3: Funnel plot of intervention versus control on LMS**

**
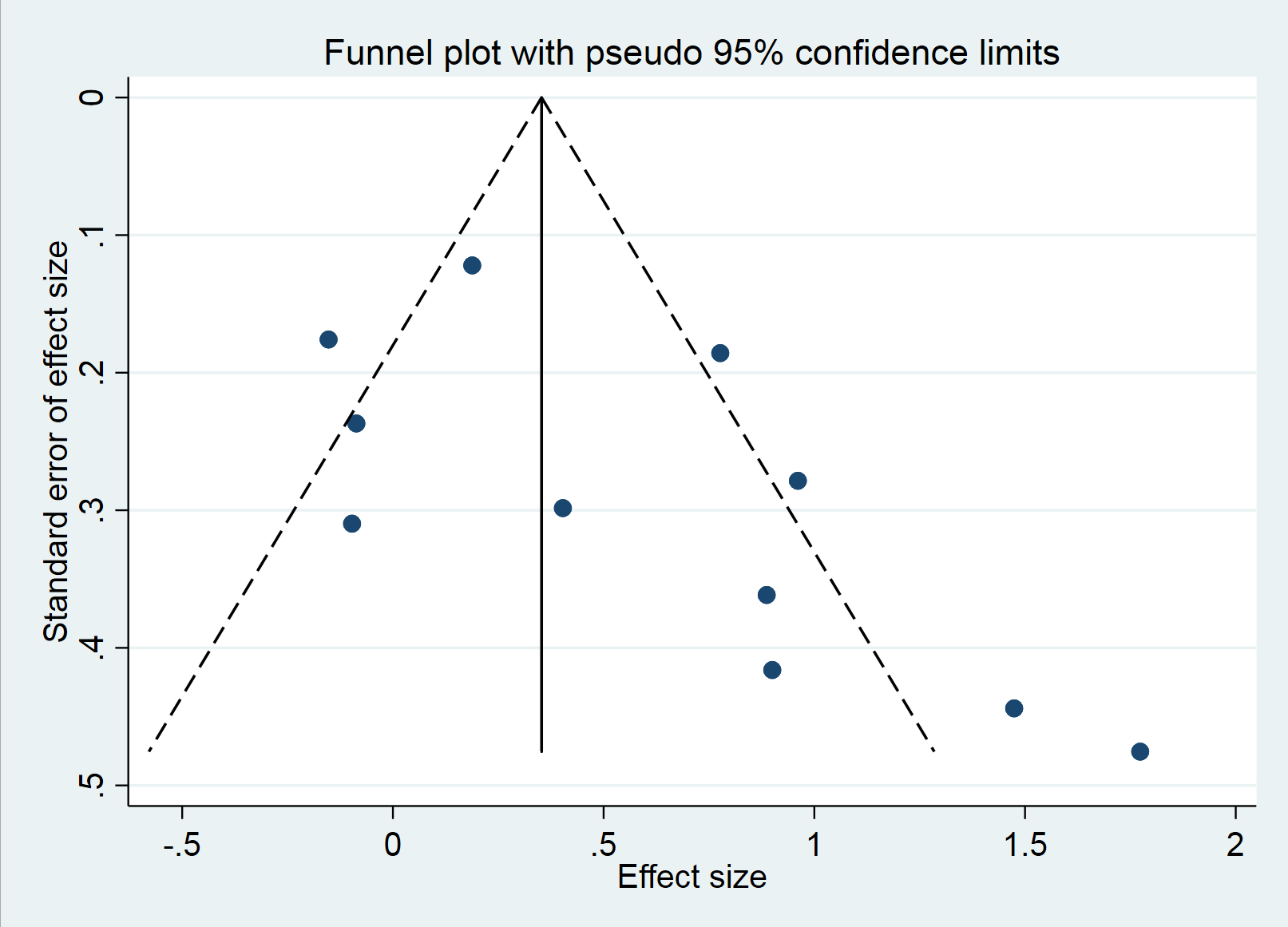
**

**Electronic Supplementary Material Figure S4: Funnel plot of intervention versus control on OCS**
